# A “sweet” way to increase the metabolic activity and migratory response of cells associated with wound healing: deoxy-sugar incorporated polymer fibres as a bioactive wound patch

**DOI:** 10.3906/biy-2108-27

**Published:** 2021-11-02

**Authors:** Serkan DİKİCİ

**Affiliations:** Department of Bioengineering, Faculty of Engineering, Izmir Institute of Technology, İzmir, Turkey

**Keywords:** 2-deoxy-D-ribose (2dDR), deoxy-sugar, vascular endothelial growth factor (VEGF), angiogenesis, wound healing, electrospinning

## Abstract

The selection of a wound dressing is crucial for successful wound management. Conventional dressings are preferable for the treatment of simple wounds. However, a bioactive wound dressing that supports wound management and accelerates the healing process is required when it comes to treating non-self-healing wounds.

2-deoxy-D-ribose (2dDR) is a small deoxy sugar that naturally occurs in human body. Although we have previously demonstrated that 2dDR can be used to induce neovascularisation and accelerates wound healing in vitro and in vivo, the literature on small sugars is conflicting, and the knowledge on how 2dDR achieves its biological activity is very limited.

In this study, several small sugars including D-glucose (DG), 2-deoxy-D-glucose (2dDG), 2deoxy-L-ribose (2dLR) were compared to 2dDR by investigating their effects on the metabolic activities of both human dermal microvascular endothelial cells (HDMECs) and human dermal fibroblasts (HDFs). Then, for the first time, a two-dimensional (2D) scratch wound healing model was used to explore the migratory response of HDFs in response to 2dDR treatment. Finally, 2dDR was incorporated into Poly(3-hydroxybutyrate-co-3-hydroxyvalerate) (PHBV) polymer fibres via electrospinning, and the metabolic activity of both types of cells in vitro was investigated in response to sugar release via Alamar Blue assay.

The results demonstrated that 2dDR was the only sugar, among others, that enhances the metabolic activity of both HDMECs and HDFs and the migratory response of HDFs in a 2D scratch assay in a dose-dependent manner. In addition to direct administration, 2dDR was also found to increase the metabolic activity of HDMECs and HDFs over 7 days when released from polymer fibres. It is concluded that 2dDR is a potential pro-angiogenic agent that has a positive impact not only on endothelial cells but also fibroblasts, which take a key role in wound healing. It could easily be introduced into polymeric scaffolds to be released quickly to enhance the metabolic activity and the migratory response of cells that are associated with angiogenesis and wound healing.

## 1. Introduction

Skin plays an important role in the prevention of the body against microorganism invasion (Lee et al., 2006), from dehydration, mechanical, chemical, and thermal damage, and exposure to ultraviolet (UV) (Forslind et al., 1997; Rees, 1999). Skin is composed of epidermal, dermal, and subcutaneous layers (Honari, 2017), and wounding could be superficial to include only the epidermal layer or deeper and more complex, including dermal and subdermal layers of the skin (Stroncek and Reichert, 2007).

Regular healing steps of a superficial wound includes six main stages: (i) haemostasis, (ii) inflammation, (iii) granular tissue formation, (iv) proliferation, (v) neovascularisation, and (vi) remodelling (Kawasumi et al., 2013). However, some of the wounds cannot go through all these steps correctly due to several reasons, such as depth and complexity of the wounds, repetitive trauma, infection, comorbidity of the patients (Han and Ceilley, 2017). In the treatment of wounds, the selection of a wound dressing is a critical factor that affects the healing process. Conventional dressings such as gauze dressings, plasters or bandages are cost-effective and useful for the treatment of simple wounds, but they are only passive dressings and do not improve the healing process. On the other hand, modern dressings such as film, foam, hydrogel, hydrocolloid, alginate dressings offer a chance to enhance the healing of the wound rather than just to cover it (Dhivya et al., 2015). These two types of dressings offer an opportunity to treat several types of wounds. However, when it comes to treating non-self-healing chronic wounds, more complex bioactive wound dressings are required (Han and Ceilley, 2017).

The physiological tissue/wound healing process is strictly controlled by cell-cell and cell-extracellular matrix (ECM) interactions (Dikici, Aldemir Dikici, et al., 2021). As a result of these interactions, growth factors are released and play a key role in stimulating angiogenesis and accelerating wound healing. Exogenous use of the wound-related growth factors facilitates the healing process (Badiu et al., 2011). The rationale behind the use of growth factors is the improvement of the chemotactic activity and proliferation of wound-related cells and increasing angiogenesis in the wound area. Among these, angiogenesis is crucial and takes a role not only in the oxygen and nutrient transport and waste removal during the very challenging proliferative phase but also in the transmigration of immune cells to the wound area during the inflammation phase. These critical tasks make angiogenesis an indispensable step for the natural healing process of wound healing (Tonnesen et al., 2000).

Platelet-derived growth factor (PDGF), fibroblast growth factor (FGF), epidermal growth factor (EGF), and vascular endothelial growth factor (VEGF) are the commonly used growth factors either to induce angiogenesis or to improve wound healing (Hunt et al., 2000). Growth factors are considered the most effective approach in promoting angiogenesis and accelerating wound healing. However, the use of growth factors is costly, and uncontrolled use of them is either ineffective or not safe (Cheng et al., 1997; Grazul-Bilska et al., 2003). VEGF has been shown to be the most effective growth factor to induce angiogenesis (Hoeben et al., 2004), but it has also been reported that the use of exogenous VEGF in an uncontrolled manner may lead to the formation of leaky, highly permeable and haemorrhagic blood vessels, as observed in tumorigenesis (Cheng et al., 1997; Yancopoulos et al., 2000; Cao et al., 2004). Other growth factors that are frequently used for wound healing, EGF, FGF, and PDGF, require either continuous and repeated applications or a carrier system in order to provide efficient healing. In addition, their high cost and safety concerns limit the clinical accessibility of these agents (Grazul-Bilska et al., 2003). Given both their high cost and the risk of tumour formation, the safety of clinical use of these pro-angiogenic agents exogenously is questionable, and the need for alternative approaches to stimulate these growth factors indirectly may offer a promising alternative for the effective and safe stimulation of angiogenesis and accelerate wound healing.

**Figure 1:**
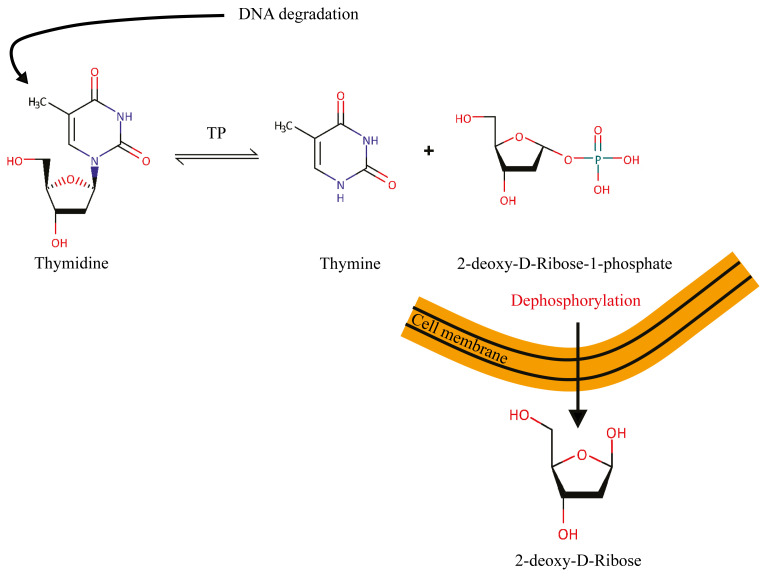
The degradation of thymidine to thymine by the enzymatic activity of TP. Following the degradation, 2dDR-1-phosphate (2dDR1P) occurs, and the dephosphorylation of 2dDR1P forms 2dDR, which can pass the cell membrane.

2-deoxy-D-ribose (2dDR) is a deoxy pentose sugar that is found in the DNA’s nucleic acid structure in the human body. It is an endogenous metabolite and naturally formed as a result of the degradation of thymidine to thymine by thymidine phosphorylase (TP) (Figure 1) (Priestle and Paris, 1996). The angiogenic potential of 2dDR was first investigated in the early 90s (Finnis et al., 1993) and then in the early 2000s (Sengupta et al., 2003). Both studies hypothesised that 2dDR as the side product might be the potential cause of the pro-angiogenic activity of the TP (today also known as platelet-derived endothelial cell growth factor (PD-ECGF)). Although these studies suggested the positive impact of 2dDR on proliferation and migration of endothelial cells, the biological and molecular efficiency in angiogenesis and wound healing processes has not been investigated in detail since then. Recently, we reported that 2dDR achieves its activity via stimulation of VEGF production by human aortic endothelial cells (HAECs) in vitro (Dikici, Aldemir Dikici, et al., 2019; Dikici, Bullock, et al., 2020) and in chick chorioallantoic membrane (CAM) assay (Dikici, Mangir, et al., 2019) in vivo. Furthermore, we demonstrated that 2dDR releasing alginate dressings is an effective treatment for chronic wounds in a diabetic rat model (Andleeb et al., 2020; Dikici, Yar, et al., 2021).

Alongside 2dDR, three other small sugars (2-deoxy-L-ribose (2dLR), 2-deoxy-D-glucose (2dDG), and D-glucose (DG)) were tested for their potential pro-angiogenic activity. These sugars were selected initially due to being involved in angiogenesis-related studies (either in a positive or negative way) in the literature. However, the rationale behind selecting (i) 2dLR was to investigate whether this effect is structure-dependent or not as 2dLR is the stereoisomer of 2dDR and to explore how conformational change affects the biological activity of a sugar, (ii) DG was to see if the effect is due to the metabolisation of sugar and again if it is independent of the sugar-origin or not, and (iii) 2dDG was to see what would happen if we use a sugar which recapitulates the function of DG but cannot undergo further glycolysis.

Wound dressings are made of both synthetic and natural materials. Natural materials are advantageous due to their superior biological activity in supporting the adhesion and functionality of cells. However, all bioactive components should be removed to avoid undesired host response upon implantation (Mano et al., 2007; Guo and Ma, 2014). On the other hand, synthetic materials are attractive due to having high control on mechanical and physical properties and no host response concerns due to their nonbiological origin (Magnusson et al., 2011).

To date, various synthetic polymers have been reported as biomaterials for their use in angiogenesis, tissue engineering, and wound healing research. These polymers include but not limited to polycaprolactone (PCL) (Zhang et al., 2007; Pektok et al., 2008; Singh et al., 2011), polylactic acid (Hegen et al., 2011; Jia et al., 2013), poly(glycolic acid) (Hoerstrup et al., 2006), poly(lactic-co-glycolic acid) (Iwai et al., 2004), polyurethane (Grasl et al., 2010; Huang et al., 2011), polyglycerol sebacate (Motlagh et al., 2006). Similar to synthetic polymers, naturally-sourced polymers are also widely used in various studies. The most popular ones are polyhydroxyalkanoates, including Poly(3-hydroxybutyrate) (Akaraonye et al., 2016), Poly(3-hydroxyoctanoate) (Bagdadi et al., 2018), Poly(3-hydroxybutyrate-co-3-hydroxyvalerate) (PHBV) (Sultana and Khan, 2012; Ortega et al., 2015), and others such as collagen (Berglund et al., 2004; Zhu et al., 2009, 2014), gelatin (Doi et al., 2007; Panzavolta et al., 2011), alginate (Augst et al., 2006; Azam et al., 2019), elastin (Engbers-Buijtenhuijs et al., 2006), silk fibroin (Lovett et al., 2010; Marelli et al., 2012), dextran (Sun et al., 2011), and hyaluronic acid (Zhu et al., 2014). Considering the pros and cons of these natural polymers, PHBV appears to have great potential as a biomaterial because of its high biocompatibility and being safely biodegradable. 3-hydroxybutanoic acid naturally occurs in human body and is a degradation product of PHBV. Its degradation product being a natural metabolite accounts for its high biocompatibility (Quillaguamán et al., 2010). In addition, PHBV offers superior mechanical strength compared to other natural polymers, and the ease of manufacturing PHBV micro and nanofibres via electrospinning makes it a good candidate for the production of fibrous bioactive wound dressings to be used in wound management (Dikici, Claeyssens, et al., 2020a).

Accordingly, in this study, the effect of different doses of 2dDR on the metabolic activity of human dermal microvascular endothelial cells (HDMECs) that has a key role in wound healing (Tonnesen et al., 2000) was evaluated compared to other small sugars in order to clarify the conflicting literature on small sugars and the specificity of the pro-angiogenic response to 2dDR. Then, the stimulation of proliferation and migration behaviour of human dermal fibroblasts (HDFs) in response to 2dDR was investigated using a two-dimensional (2D) wound healing (scratch) model. Following the dose-response and 2D wound healing studies, 2dDR was incorporated into PHBV nanofibres to be used as a bioactive wound dressing and showed its release over 10 days. 2dDR-releasing PHBV dressings were then evaluated for their performance in improving the viability of two different types of cells, HDFs and HDMECs, both of which actively take part in the wound healing process in vivo.

## 2. Materials

A total of 37% formaldehyde solution, AlamarBlue cell viability assay, amphotericin, penicillin/streptomycin, dimethyl sulphoxide (DMSO), dichloromethane (DCM), methanol, fetal bovine serum (FBS), vascular endothelial growth factor (VEGF), epithelial growth factor (EGF), phosphate-buffered saline (PBS), Poly3-hydroxybutyrate-co-3-hydroxyvalerate (PHBV), and trypsin EDTA were purchased from Sigma Aldrich. Human Dermal Microvascular Endothelial Cells (HDMECs), Endothelial Cell Growth Medium (EC GM) and EC GM Supplement Pack were purchased from PromoCell. Human Dermal Fibroblasts (HDFs) and Fibroblast Growth Medium (FGM) BulletKit were purchased from Lonza.

## 3. Methods

### 3.1. Assessment of the metabolic activity of HDMECs in response to small sugars (2dDR, 2dLR, 2dDG, DG) and VEGF

A total of 10 mM stock solutions of 2dDR, 2dLR, 2dDG and DG were prepared by dissolving the sugars in low serum (2% FCS) EC GM and filtered using a 0.2 μm syringe filter. The stock sugar solutions were then diluted 10× and 100× in sterile EC GM to prepare 1 mM and 100 μM sugar solutions. 80 ng/mL VEGF solution was prepared in sterile EC GM and used as a positive control. Low serum EC GM was used as a control group in the metabolic activity experiments.

HDMECs, between passages 2 and 6, were cultured in EC GM supplemented with a total of 10% FBS and growth medium 2 kit supplements at 37 °C and 5% CO_2_. The growth medium was replaced every 3 days until they reached ~80%–90% confluency.

AlamarBlue Cell Viability Assay was used to measure the metabolic activity of HDMECs over 7 days in response to 100 μM, 1 mM and 10 mM concentrations of 2dDR, 2dLR, 2dDG, and DG as described previously (Aldemir Dikici, Sherborne, et al., 2019; Dikici, Claeyssens, et al., 2019). Once HDMECs reached confluence, they were collected, prepared into a cell suspension, and added into 48-well plates (seeding density is 1×10^4^ cells/cm^2^) HDMECs were then cultured with EC GM either supplemented with 100 μM, 1 mM and 10 mM concentrations of 2dDR, 2dLR, 2dDG, DG, or VEGF or as non-supplemented (control). AlamarBlue readings were taken on days 1, 4, and 7 to track the metabolic activity changes in response to sugar treatments. Briefly, 0.1 mM AlamarBlue solution was prepared in EC GM. The growth medium of cells was removed, and the cells were washed with PBS once. Then, 1 mL of AlamarBlue solution was added to each well and incubated at 37°C for 4 h. After the incubation, 200 μL of the solution was transferred into a 96-well plate, and the fluorescence readings were taken at 540 nm (excitation) and 635 nm (emission).

### 3.2. The effect of 2dDR on the metabolic activity of human dermal fibroblasts (HDFs)

Following the evaluations of different small sugars’ effects on metabolic activity change of HDMECs, further doses of 2dDR (1 μM, 100 μM, 1 mM, 10 mM) were assessed to see the effect of it on increasing the metabolic activity of HDFs. For this, HDFs, between passages 3 and 8, were cultured in FGM supplemented with a total of 10% FBS and BulletKit supplements at 37°C and 5% CO_2_. The growth medium was replaced every 3 days until they reached ~80%–90% confluency.

AlamarBlue Cell Viability Assay was used to measure the metabolic activity of HDFs over 7 days in response to 1 μM, 100 μM, 1 mM, and 10 mM concentrations of 2dDR. Once HDFs reached confluence, they were collected, prepared into a cell suspension, and added into 48-well plates (seeding density is 1×10^4^ cells/cm^2^). HDFs were cultured with FGM either supplemented with 1 μM, 100 μM, 1 mM, and 10 mM of 2dDR or non-supplemented (control). AlamarBlue readings were taken on days 1, 4, and 7 to track the metabolic activity changes in response to sugar treatments as described in Section 3.1.

### 3.3. Evaluating the effect of 2dDR on HDFs using a 2D scratch wound healing assay

Scratch wound healing assay (Liang et al., 2007) was used to evaluate the migratory response of HDFs when administered with 2dDR compared to positive control EGF. The principle of this assay is the destruction of confluent cell monolayer to generate an acellular region, which is available to cells for migration and repair. For this, HDFs were cultured as described above, trypsinised, and seeded into 24-well plates with a seeding density of 2×10^4^ cells/cm^2^. Once HDFs are confluent, a scratch wound was created by scraping the cell monolayer in a straight line using a 200 μL pipette tip. Caution was given to keep the sizes and width of the wound areas similar to minimise variations.

Following the scratch, well plates were gently washed with PBS to remove detached cells. Then, the FGM (with antibiotics and 5% FBS) was replenished either supplemented with 2dDR (1 μM and 1 mM), EGF (50 ng/mL) or as non-supplemented (control). The well-plates were returned to a cell culture incubator and incubated at 37 °C, 5% CO_2_, for 60 h until a full closure was observed in one of the groups.

An inverted microscope (Olympus, Japan) was used to take digital images of the wound area. The images were taken at the beginning of the experiment and after 12, 48, and 60 h. Adobe Photoshop CS6 software (ADOBE Systems Inc., San Jose, California, USA) was used to convert the raw image to a black and white image to facilitate image processing. For this, wound areas were drawn manually by the magic wand (quick selection) tool. The areas outside the wound zone were coloured black, while the wound zone was coloured with white. Black and white images were then imported to ImageJ software (Wayne Rasband, National Institutes of Health, USA). The white zones corresponding to the wound area were quantified using the histogram function of ImageJ software, where histograms of each image were analysed, and the white areas in the histograms were tabulated to Microsoft Excel software to calculate the percentage of wound reduction for each group. Normalised percentage wound closure rates were determined as the percentage wound area reduction per the time point that the image was acquired.

### 3.4. Manufacturing of the PHBV nanofibrous wound dressings loaded with 2dDR

Electrospinning was used to fabricate 2dDR releasing PHBV fibres as bioactive wound dressings (Dikici, Mangir, et al., 2019; Dikici, Claeyssens, et al., 2020b). Briefly, 10% (w/w) electrospinning solution was prepared by dissolving PHBV in DCM:methanol (90:10 w/w) in a fume hood prior to electrospinning. 100 mg and 250 mg of 2dDR per 1 g of PHBV were added to the electrospinning solution prior to electrospinning. 5 mL of the solutions were then loaded into 10 mL syringes equipped with 0.6 mm blunt syringe tips and placed in a syringe pump. The electrospinning was performed at room temperature from a fixed collector distance of 17 cm with a flow rate of 40 μL/min, and a voltage of 17 kV until all the solution was exhausted.

The surface morphology of 2dDR releasing wound dressings was investigated under a scanning electron microscope (SEM) (FEI QUANTA 250 FEG, USA) (Aldemir Dikici et al., 2020). Fibre diameter and pore sizes were measured using ImageJ as described previously (Aldemir Dikici, Dikici, et al., 2019; Dikici, Claeyssens, et al., 2020a).

### 3.5. Assessment of 2dDR release from the PHBV fibres

A spectrophotometric method was used for the detection of 2dDR, as described previously (Dikici, Mangir, et al., 2019; Dikici, Bullock, et al., 2020 ). Briefly, 100 mg and 250 mg 2dDR loaded PHBV dressings were cut into pieces so to fit into a 6-well plate, weighed before the release experiments and incubated in 2 mL of PBS for 10 days. The 2dDR release were measured fluorometrically using a spectrophotometer (Shimadzu UV2550, Japan). The readings were converted to a percentage release of 2dDR from the dressings before reporting.

### 3.6. Evaluation of the in vitro biological performance of 2dDR releasing PHBV dressings

Following the fabrication of 2dDR releasing PHBV dressings, they were cut into 10 mm diameter circles using a biopsy punch and disinfected in 70% ethanol for 45 min. Then, the dressings were washed in PBS for 1 h (PBS was refreshed every 20 minutes for a total of three times) and placed into 12-well plates for cell seeding.

HDMECs and HDFs were cultured as described in the previous sections, and once they reached to confluence, they were trypsinised and seeded onto 2dDR releasing PHBV scaffolds with a seeding density of 4 × 10^4^ cells/cm^2^. Scaffolds were kept at 37 °C for 60 min to allow cells to attach. Then, 4 mL of EC GM (for HDMECs) and FGM (for HDFs) were added to each well. PHBV dressings were kept in culture for 7 days by changing the culture medium every 2–3 days.

The metabolic activity of HDMECs and HDFs seeded onto 2dDR releasing PHBV fibres were tracked over 7 days using Alamar Blue Cell Viability Assay as described in previous sections (Dikici, Claeyssens, et al., 2020a). The metabolic activity measurements were taken on days 1, 4, and 7 as described in Section 3.1.

### 2.7. Statistical analysis

Statistical analysis was performed using a statistical analysis software (GraphPad Prism, California, USA). The analysis was conducted either using one-way or two-way analysis of variance (ANOVA). Error bars indicate standard deviations unless otherwise stated.

## 4. Results

### 4.1. 2dDR improves the metabolic activity of HDMECs but no other small sugars, 2dLR, 2dDG, and DG

The results of the AlamarBlue assay demonstrated that 2dDR, 2dLR and DG did not cause any change in the metabolic activity of HDMECs compared to controls at any doses tested, while VEGF increased the metabolic activity significantly. In contrast, 2dDG at 1 mM and 10 mM doses was found to decrease the cellular activity even on day 1.

**Figure 2:**
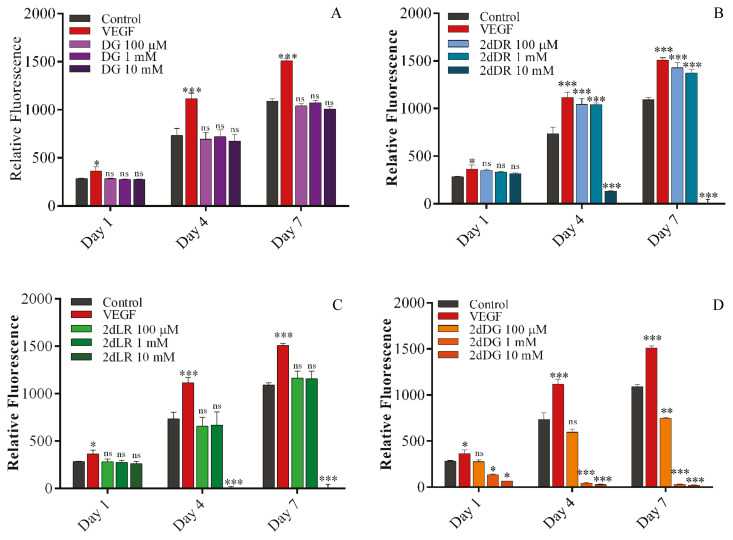
The metabolic activity changes of HDMECs in response to treatment of several small sugars. The results of the AlamarBlue metabolic activity assay in response to 100 μM, 1 mM, 10 mM concentrations of (A) DG, (B) 2dDR, (C) 2dLR, and (D) 2dDG treatment (***p ≤ 0.001, **p ≤ 0.01, *p ≤ 0.05, not significant (ns) p ≥ 0.05, n = 3 ± SD).

2dDR at 100 μM and 1 mM doses and VEGF at 80 ng/mL (as the positive control) has been found effective in terms of increasing the activity of HDMECs on days 4 and 7. In contrast to 100 μM and 1 mM doses, the highest concentration (10 mM) of 2dDR was found to decrease the activity significantly on days 4 and 7. A similar trend was observed for 2dLR at 10 mM dose, whereas lower doses (100 μM and 1 mM) were ineffective at any time points, and no statistically meaningful change was observed compared to controls. For 2dDG, the reductions in the metabolic activity of HDMECs on day 4 and 7 were more dramatic compared to 2dDR and 2dLR, including the mid-dose (1 mM) alongside the highest dose of 10 mM. However, 100 μM dose of 2dDG caused a significant reduction in the activity only on day 7 but not on days 1 and 4 (no significant change was observed compared to controls). Throughout the metabolic activity measurements, DG did not show any change in the metabolic activity of HDMECs at any concentration on any days compared to controls. The metabolic activity changes of HDMECs over 7 days in response to 100 μM, 1 mM, and 10 mM doses of DG (Figure 2A), 2dDR (Figure 2B), 2dLR (Figure 2C), and 2dDG (Figure 2D) treatments are given in Figure 2.

Take home messages from this sugar comparison study were as follows: (i) 2dDR (at 100 μM and 1 mM) was the only sugar that enhances the metabolic activity of HDMECs significantly at the end of 7 days, (ii) 2dDR (at 10 mM, on days 4 and 7), 2dLR (at 10 mM, on days 4 and 7) and 2dDG (at 100 μM, 1 mM and 10 mM, on days 1, 4 and 7) caused a dramatic decrease in the activity of HDMECs due to probable dose-dependent toxicity. For 2dDG, this may not be surprising be explained as 2dDG has previously been demonstrated as a potential anti-angiogenic agent (Merchan et al., 2010; Kovacs et al., 2016). However, future studies to investigate the negative effect of 2dDR and 2dLR at higher concentrations on cells may provide significant knowledge on the safety and potential dose-dependent anti-angiogenic activity of these sugars.

### 4.2. 2dDR increases the metabolic activity of HDFs over 7 days

**Figure 3:**
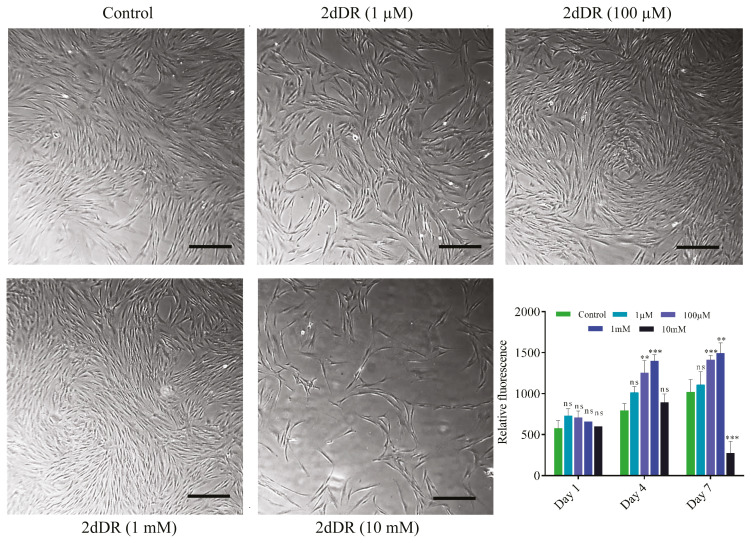
Light microscope images show the morphologies of HDFs at the end of day 7. Graph shows the results of the AlamarBlue metabolic activity assay in response to 2dDR treatment over 7 days. (***p ≤ 0.001, **p ≤ 0.01, not significant (ns) p ≥ 0.05, n = 3 ± SD). Scale bars represent 200 μm.

The metabolic activity measurements of HDFs over 7 days showed that similar to that observed on HDMECs, 2dDR treatment at 100 μM and 1 mM doses also increased the metabolic activity of HDFs by days 4 and 7, while there was no statistically meaningful difference on day 1. Although 1 μM dose of 2dDR caused a slight increase by days 4 and 7, it was not statistically different compared to controls. The light microscope images of HDFs at the end of day 7 and the results of the AlamarBlue metabolic activity assay in response to 2dDR treatment over 7 days are given in Figure 3.

One thing to note is that, similar to HDMECs, a significant decrease was observed in the activity of HDFs when treated with 10 mM 2dDR. However, it seems like HDFs were more tolerable to the highest dose of 2dDR as the rate of decrease was lower compared to that of HDMECs. In addition, light microscope images also revealed that there were still some HDFs attached to the well plate in contrast to endothelial cells, which has previously been reported to detach completely from the well plate when treated with 10 mM of 2dDR (Dikici, Aldemir Dikici, et al., 2019).

### 4.3. 2dDR enhances the migratory response of HDFs in 2D scratch wound healing assay

2D scratch assay is a convenient and inexpensive protocol for the analysis of cell migration corresponding to wound healing in vitro (Liang et al., 2007). When assessing the cellular migration in a scratch model, analysis of two parameters provides valuable information on the migratory response: the wound closure percentage and the normalised wound closure rate. The wound closure percentage indicates the percentage repopulation of the scraped area by the cells. The normalised wound closure rate demonstrates how effective the active agent to accelerate the closure of the scraped area compared to controls (either negative or positive) (Abdel-Naby et al., 2017; Da Silva et al., 2019).

**Figure 4:**
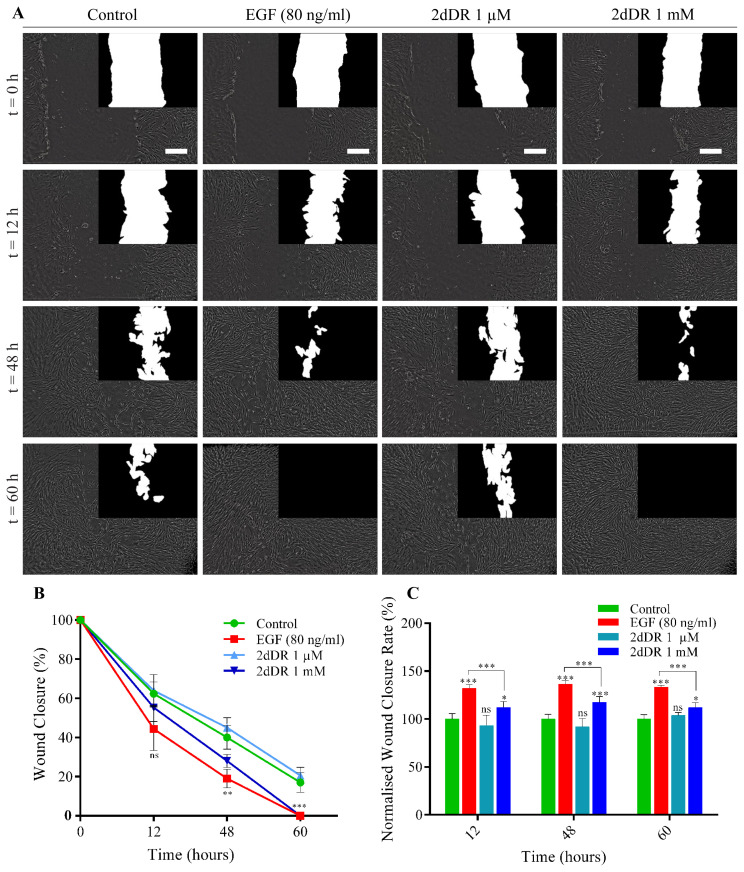
(A) The inverted light microscope and the processed images (top right of each image) showing the migratory response (corresponding to 2D wound closure over 60 hours) of HDFs when treated with two doses of 2dDR and EGF (positive control) compared to controls. Images from the same wound area were taken at 0, 12, 48, and 60 h after the wound is created. Scale bars represent 200 μm. The graphs show (B) the wound closure (%) and (C) normalised wound closure rate (%) of HDFs in response to 2dDR treatment. (***p ≤ 0.001, **p ≤ 0.01 *p ≤ 0.05, not significant (ns) p ≥ 0.05, n = 3 ± SD).

Following the metabolic activity studies of HDFs, the scratch wound healing assay also showed that 1 mM of 2dDR caused an increase in the migratory response of HDFs in 60 h compared to controls (Figure 4A). Although none of the groups, including the positive control (EGF), showed a statistically meaningful increase in the wound closure by 12 hours, EGF and 1 mM 2dDR treatment significantly accelerated the wound healing by 48 h compared to controls. Although a slight increase was observed in the percentage of wound closure, 1 μM 2dDR has not been found statistically different than controls (Figure 4B).

When ormalized wound closure rates were investigated, only EGF and 1 mM 2dDR treatments have been found to increase the closure rates by 12, 48, and 60 h but no other groups. EGF treatment increased the wound closure rate by approximately 1,3-fold at all time points (12, 48 and 60 hours). Similarly, 1 mM 2dDR treatment caused an increase of 1.1-fold, 1.2-fold and 1.1-fold by 12, 48, and 60 h, respectively, in the wound closure rates (Figure 4C).

The light microscope and the processed images (given in the top right of each image) showing the migration of HDFs corresponding to 2D scratch wound closure over 60 h, and the quantified graphs of percentage wound closure and normalised wound closure rates are given in Figure 4.

### 4.4. The results of the fabrication of 2dDR releasing PHBV wound dressings and the release of 2dDR from the fibres over 10 days

**Figure 5:**
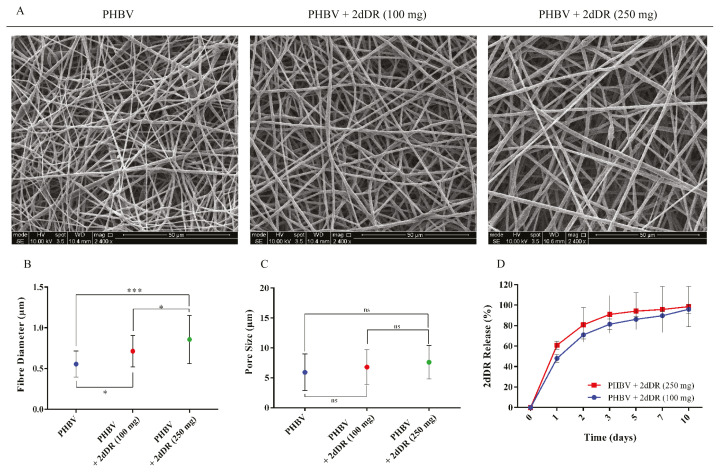
(A) The SEM images, (B) average fibre diameter, (C) average pore sizes of the PHBV electrospun wound dressings. (D) The graph shows the release behaviour of 2dDR over 10 days from the PHBV fibres. (***p ≤ 0.001, *p ≤ 0.05, not significant (ns) p ≥ 0.05, n = 3 ± SD).

SEM images of the 2dDR releasing PHBV dressings are given in Figure 5A. The addition of both 2dDR concentrations significantly increased the fibre diameters of the electrospun PHBV dressing. The inclusion of 100 mg and 250 mg of 2dDR in the PHBV electrospinning solution resulted in an increase in the average fibre diameter from 0.55 ± 0.16 μm (control) to 0.71 ± 0.19 μm and 0.86 ± 0.29 μm, respectively. In contrast to fibre diameters, no statistically meaningful increase was observed in the pore sizes when 2dDR was added. The average pore sizes were calculated as 5.92 ± 3.07 μm, 6.79 ± 2.90 μm, and 7.60 ± 2.79 μm, respectively for PHBV, PHBV + 2dDR (100 mg) and PHBV + 2dDR (250 mg). The average fibre diameter and pore sizes were shown in Figure 5B and 5C, respectively.

The releases of 2dDR from PHBV fibres was measured over 10 days, as shown in Figure 5D. By 3 days, 2dDR releases from the PHBV fibres were approximately 82.2% and 90.6% of the total 2dDR present in the polymer solution for 100 mg and 250 mg 2dDR loaded scaffolds, respectively. Almost 90.5% and 95.8% of the total 2dDR were released from the 100 mg and 250 mg 2dDR loaded dressings, respectively, by 7 days. No statistical difference was found between the released amounts of 2dDR from the dressings over 10 days, and, considering the possible negative effect of the higher doses of 2dDR on cells, such as observed in metabolic activity studies, 100 mg 2dDR loaded PHBV dressings were selected for further in vitro study.

### 3.5. 2dDR releasing wound dressings improves the metabolic activity of HDMECs and HDFs in vitro

**Figure 6:**
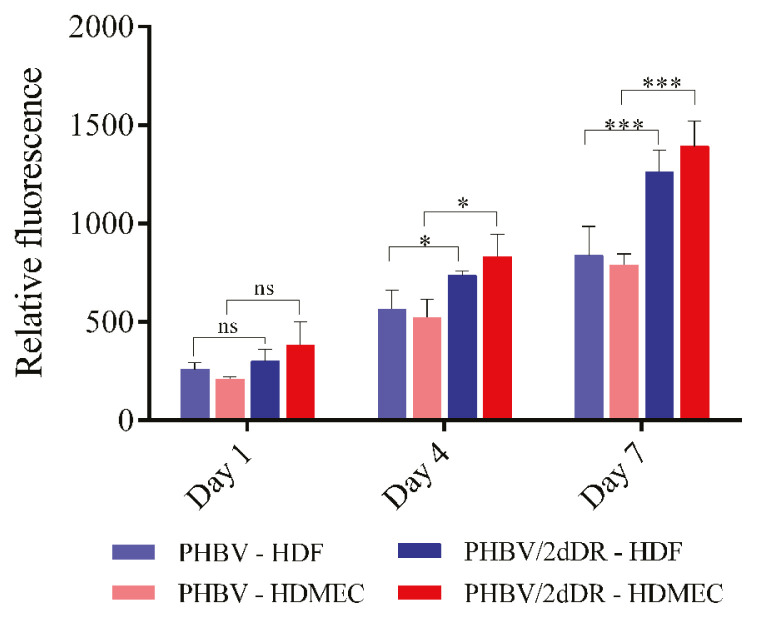
The metabolic activity of HDFs and HDMECs growing on the 2dDR releasing PHBV dressings over 7 days. (***p ≤ 0.001, *p ≤ 0.05, not significant (ns) p ≥ 0.05, n = 3 ± SD).

Alongside the direct addition of 2dDR to the growth media, the 2dDR released from the PHBV dressings were also found to increase the metabolic activity of HDMECs and HDFs over 7 days (Figure 6).

By day 1, the metabolic activity of HDFs was higher than that of HDMECs attached to plain PHBV dressing. Although there was no statistically meaningful difference, the activity of HDMEC and HDFs went up by 1.6-fold and 1.1-fold, respectively, when seeded onto 2dDR releasing PHBV dressings. On day 4, by the release of 2dDR from the PHBV dressings, the metabolic activity of HDMECs and HDFs were increased significantly by approximately 1.6-fold and 1.3-fold, respectively. By day 7, cell activity went up by 1.5-fold and 1.8-fold for HDFs and HDMECs seeded onto the 2dDR-releasing PHBV fibres, respectively.

## 4. Discussion

Acute wounds can follow the physiological healing steps successfully and heal with an expected rate and within a predicted time. However, chronic, deeper and complex wounds where usually more than one layer of skin is damaged, and a constant infection occurs due to delayed healing requires special attention and a bioactive approach for successful healing.

**Figure 7:**
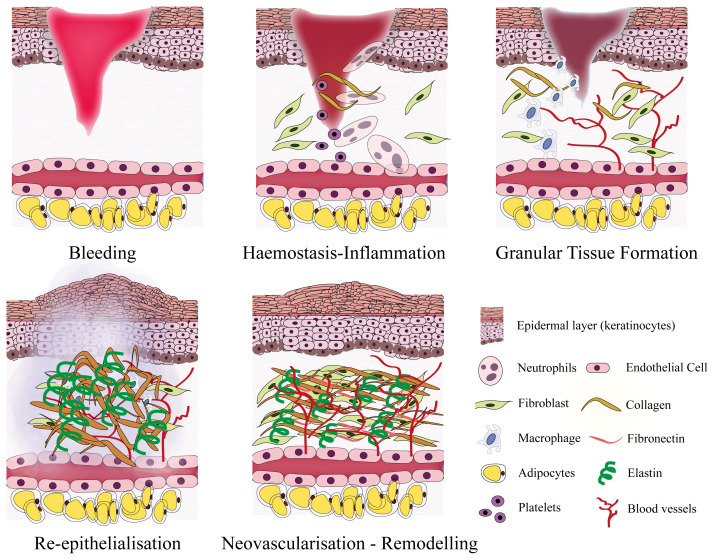
Schematic illustration of wound healing process. Six main phases of wound healing (bleeding, haemostasis, inflammation, granular tissue formation, reepithelialisation, neovascularisation, and remodeling) were explained considering changes in cellular densities and activities, extracellular matrix structure and density, and neovascularisation in the wound area.

Physiological wound healing is a complicated process where cell-cell, cell-ECM interactions and growth factors play key roles. The main steps of wound healing are haemostasis, inflammation, granular tissue formation, proliferation, neovascularization, and remodelling (Figure 7) (Kawasumi et al., 2013). Ensuring a rapid and successful vascularisation in the wound healing are is vital particularly in the management of complex and chronic wounds that fail to follow the physiological healing steps without further assistance (Honnegowda et al., 2015). Neovascularisation is an indispensable step of the wound healing, and it plays a pivotal role not only in the proliferative phase by supplying oxygen and nutrients and by removing the waste and debris but also in the transmigration of immune cells to the wound area during the inflammation phase (Tonnesen et al., 2000).

The use of growth factors is a well-established and effective way of developing active wound patches to induce angiogenesis and accelerate wound healing. Among many growth factors, VEGF is considered the most effective stimulator of angiogenesis (Hoeben et al., 2004). However, it has also been shown to stimulate extremely leaky (Yancopoulos et al., 2000), too permeable (Cao et al., 2004), and haemorrhagic (Cheng et al., 1997) vessels that correspond to tumour angiogenesis (Oka et al., 2007). Other growth factors such as EGF, FGF, and PDGF are frequently used to accelerate wound healing ( Badiu et al., 2011; Servold, 1991). However, most of the growth factors are too expensive to be used with traditional wound dressing manufacturing methods. They are also not very stable, require highly sensitive carrier systems due to narrow effective dose ranges, and possess safety concerns that limit the clinical accessibility of these agents (Grazul-Bilska et al., 2003). Given both their high cost and the risk of tumour formation, the safety of clinical use of pro-angiogenic agents is questionable, and the need for the investigation of alternative factors to stimulate angiogenesis and accelerate wound healing is clear.

2dDR is a small deoxy pentose sugar that is naturally found in the human body in the nucleic acid structure. During the dynamic metabolic processes of the DNA, 2dDR is formed by the TP activity as a side product of the enzymatic degradation of thymidine into thymine (Priestle and Paris, 1996). Starting from the early 90s (Finnis et al., 1993; Sengupta et al., 2003), 2dDR has gained attention as the main cause of the pro-angiogenic activity of TP. However, none of these studies has investigated the details of the 2dDR’s pro-angiogenic potential and wound healing accelerating properties. After almost 23 years, we have recently started to investigate 2dDR as a potential proangiogenic and wound healing agent and conducted dose-response studies in vitro (Dikici, Aldemir Dikici, et al., 2019) and in vivo (Dikici, Mangir, et al., 2019)*.* In addition, we investigated its potential to accelerate the healing of chronic wounds using a diabetic rat wound healing model (Andleeb et al., 2020) and showed that its pro-angiogenic activity is via upregulating the production of VEGF by HAECs (Dikici, Bullock, et al., 2020). Although we have shown the biological efficiency of 2dDR on angiogenesis and wound healing at the organism level, the investigation of these properties at the cellular level using the cell types associated with wound healing still possess critical importance to reveal the details of the 2dDR’s activity. The main role of HDFs in angiogenesis and wound healing is to produce significant amounts of ECM molecules (Bishop et al., 1999; Sorrell et al., 2007) and growth factors (Black et al., 1998; Hudon et al., 2003). Thus, the importance of the exploration of how HDFs would react to 2dDR treatment is clear to be able to fully understand the 2dDR’s biological importance. However, to date, no study has been conducted to investigate the effect of 2dDR on fibroblasts’ proliferative and migratory responses.

Accordingly, in this study, three potential doses of 2dDR were compared with other small sugars, 2dLR, 2dDG, and DG to investigate its specificity to HDMECs, a small-diameter blood vessel endothelial cells sourced from human skin, which is a great representative cell type that can be used in vitro angiogenesis and wound healing studies (Oberringer et al., 2007). Then, the effect of 2dDR treatment on HDFs’ proliferative and migratory response was explored. Finally, 2dDR was loaded into PHBV fibres to investigate its potential to be used as a bioactive wound dressing.

2dLR, 2dDG, and DG sugars were selected not only due to being involved in angiogenesis studies in literature but also in an attempt to simply clarify whether the biological activity of 2dDR is structure dependent or is it due to the metabolism of any sugar by the cells. This allowed us to partly investigate the specificity of 2dDR as a potential pro-angiogenic agent to wound healing related cells. Our current knowledge on small sugars and how they are involved in angiogenesis pathways is quite conflicting. For instance, 2dLR has previously been reported as a promoter (Sengupta et al., 2003) and inhibitor (Uchimiya et al., 2002) of angiogenesis almost in the same years. Similarly, DG has been demonstrated as a potential agent to induce (Shigematsu et al., 1999) and reduce (Teixeira and Andrade, 1999) angiogenesis. 2dDR has previously been reported to induce apoptosis of normal human fibroblasts when used at very high concentrations (Kletsas et al., 1998) but in contrast, we have reported its positive impact on endothelial cells (Dikici, Aldemir Dikici, et al., 2019; Dikici, Bullock, et al., 2020) and HDFs (this study) when used at 100 μM to 1 mM concentrations. These examples clearly state the great importance of further exploration of dose studies of these small sugars on cell types associated with wound healing to reveal their potential therapeutic use in wound management. In this context, it is aimed to clarify these small sugars’ angiogenic and anti-angiogenic activities in a dose-dependent manner. Our results showed that 2dDR was the only one to increase the metabolic activity of HDMECs among all the small sugars used in this study. Treatment of 100 μM and 1 mM 2dDR significantly increased the cellular activity over 7 days. One probable explanation is the upregulation of VEGF in response to the 2dDR treatment of the cells (Dikici, Bullock, et al., 2020). At the higher doses (10 mM), 2dDR caused the detachment of HDMECs from the well plate such as previously observed for HAECs (Dikici, Aldemir Dikici, et al., 2019). 2dDR has also been found effective to increase fibroblast activity when administered at similar doses. 100 μM and 1 mM doses also increased the activity of HDFs, where 1 μM was not effective. One thing to note was that the highest concentration (10 mM) caused the death of HDFs, but less detachment of HDFs was observed compared to HDMECs. This is not surprising as HDMECs are quite sensitive to culture conditions than HDFs (Dikici, Claeyssens, et al., 2020b). This cytotoxic effect of 2dDR at 10 mM is likely to be due to the dose-dependent effect of 2dDR, which has previously been reported to induce apoptosis of normal human fibroblasts when used at high concentrations (10 mM to 50 mM) (Kletsas et al., 1998). The results of the 2D scratch wound healing assay demonstrated that 2dDR was increasing the migratory response of HDFs with an increased wound closure rate. One mM concentration of 2dDR has been found approximately 88% and 84% effective at 48 h and 60 h, respectively, in wound closure rate compared to EGF, positive control. At the end of 60 h, no wound closure was observed in the control groups.

2dDR is highly soluble in water and organic solvents, which makes it easy to incorporate into polymeric fibres. The addition of 2dDR into the PHBV solution did not alter the microstructure of the electrospun fibres. A smooth and beadless morphology was obtained from all PHBV electrospinning groups. Both 2dDR concentrations slightly increased the average fibre diameter of the scaffolds but did not change the average pore size. Although studying the release kinetics of 2dDR would extend our knowledge on what would be an ideal release behaviour for the acceleration of wound healing and how does the release of 2dDR from PHBV fibres fits this profile, exploration of the release kinetics of 2dDR was beyond the merits of this study. Thus, the main objective of this work was kept as simple as possible to investigate whether 2dDR can be loaded into PHBV fibres and released from them in a certain period of time to show the biological activity. The release behaviour of 2dDR from the PHBV fibres was observed over 10 days from both concentrations. More than 90% of the 2dDR was released just in 7 days. We have previously observed a similar release behaviour from a variety of biomaterials ( Azam et al., 2019; Dikici, Mangir, et al., 2019; Andleeb et al., 2020), which has been found to stimulate angiogenesis and accelerate wound healing, respectively. Similarly, in this study, we observed a significant increase in the metabolic activity of two cell types that take key roles in wound healing, HDMECs and HDFs, when cultured on 2dDR releasing PHBV dressings over 7 days compared to controls. By day 7, The release of 2dDR from PHBV dressings increased the metabolic activity of HDFs and HDMECs significantly by 1.5-fold and 1.8-fold, respectively.

To date, no studies have been conducted to investigate the effect of 2dDR on the migratory and proliferative response of fibroblast cells, which take important roles in physiological wound healing process (Desjardins-Park et al., 2018). This study makes a unique contribution to the field of angiogenesis and wound healing by exploring the stimulatory role of 2dDR on fibroblasts, for the first time, alongside endothelial cells. Furthermore, the data presented in this paper will contribute significantly to the translation of 2dDR incorporated wound management products into the clinic. Although our current knowledge (including the conclusions from this study) on 2dDR demonstrates its high potential to be used to promote angiogenesis and accelerate wound healing in vitro and in vivo, it is important to note that further investigation of the inhibitory effect of 2dDR when given at higher concentrations may prove significant information on the safety and potential dose-dependent anti-angiogenic activity prior to the clinical translation of this small sugar. Finally, the metabolism of 2dDR by bacteria localised either surrounding or within the wound is needed to be investigated to reveal whether 2dDR might lead to overgrowth of pathogenic species and consecutively wound infection.

## 5. Conclusion

To conclude, among the small sugars investigated in the scope of this study, 2dDR was the only small sugar that increases the metabolic activity of HDMECs and HDFs when used as a supplement in the growth medium. In addition, 2dDR has also been shown, for the first time, to stimulate the migratory response of HDFs in a 2D wound healing assay when used at 1 mM concentration. It has been found approximately 85% effective in terms of accelerating the wound closure rate compared to EGF, positive control. Alongside the direct addition to the growth medium, 2dDR was also effective in increasing the metabolic activity of both cell types when released from PHBV fibres over 7 days. The release of 2dDR improved the metabolic activity of HDFs and HDMECs growing on polymer fibres by 7 days. This study demonstrates that the introduction of 2dDR into the biomaterials offers a promising approach that can be used to enhance not only the metabolic activity of endothelial cells but also the metabolic activity and the migratory response of fibroblasts, which play a key role in wound healing.
